# Grand Challenges in Phage Biology

**DOI:** 10.3389/fmicb.2021.715039

**Published:** 2021-07-08

**Authors:** Sangryeol Ryu

**Affiliations:** ^1^Department of Food and Animal Biotechnology, Seoul National University, Seoul, South Korea; ^2^Department of Agricultural Biotechnology, Seoul National University, Seoul, South Korea

**Keywords:** phage (bacteriophage), phage therapy and biotechnology, host-pathogen interaction, endolysin, phage engineering

Over one century has passed since William Twort first discovered phages in 1915 and Felix d'Herelle recognized their potential as antibacterial agents in 1917 (Clokie et al., 2011). Phages were initially regarded as a silver bullet to bacterial infection before introduction of antibiotics, however, since then their use as therapeutic agents in the West tumbled because antibiotics are more potent and easier to use. Phages, mainly a few Escherichia coli phages, were then used as a model system to study fundamentals of genetics and molecular biology because they normally have simple life cycle and relatively small genome. For several decades we saw continually declining research activity on phage biology; but their enormous number, extremely high diversity, and their ubiquitous presence on earth revealed by molecular studies including viral metagenomics (Dion et al., 2020, p. 17) along with the global emergence of antibiotics resistant bacteria have renewed our interest in bacteriophages. The number of complete phage genomes listed in the NCBI Nucleotide database was 8,437 on September 2019 (Dion et al., 2020) but the number more than doubled to 17,630 as of May 2021. Even with their relatively small genome, it is becoming evident that the impact of phage on evolution and virulence development of prokaryotic cells and eventually on surrounding ecosystem is far bigger than originally thought. Genome analysis of phages has revealed that more than half of the phage genes have low or no homology to any genes on databases, suggesting that our knowledge about phage biology is just the tip of the iceberg and that the phage genome is a rich source of novel genes for biotechnology. However, there are conflicting reports about the utilization of phages for biotechnology, especially on the impact of the host immune response to phage therapy (Zaczek et al., 2016; Sweere et al., 2019). This is an important issue and needs to be properly addressed and investigated to design advanced and safe phage therapy. Overall, grand challenges of phage research are how to incorporate multidisciplinary approaches including modern molecular and microbial research to get a comprehensive understanding of novel genetic and physiological traits of phage, phage-bacteria interactions, and phage resistance.

## Host-Phage Interactions

The coevolution between bacteria and bacteriophages plays a key role in driving and maintaining microbial diversity. Research into bacteria–phage coevolution in various ecosystems is beginning to unravel factors that drive bacterial diversification based on the diversity of elements in the environments. One example of the coevolutionary dynamics in bacteria–phage interaction affected by the environment is phage-resistance development in *Salmonella* is slower when *Salmonella* and phage are cocultured on the food matrix than in the liquid culture. Recent advances in sequencing and omics technologies such as metagenomics, transcriptomics, and proteomics are providing new and much needed dimensions in analysis of these complex interactions between phages and their bacterial hosts (Salazar et al., 2021). This knowledge about mechanisms of phage resistance of the host bacteria is extremely valuable when applying to phage therapy to minimize phage resistance or the fermentation industry where phage-resistant strains are required (O'Sullivan et al., 2019).

Another element in the coevolution between bacteria and phage is bacteria and archaea have developed multiple sophisticated lines of active defense strategies including restriction-modification (R-M) systems, CRISPR-Cas, abortive infection systems (Abi), adsorption inhibition (A-I), and neutralizing bacteriophage exclusion (BREX), prokaryotic Argonautes (pAgos), and DISARM (Doron et al., 2018; Hampton et al., 2020). Considering the massive diversity of phage, unknown phage defense systems of bacteria are still waiting to be found and can be employed for great advancements. However, a challenge in host-phage interaction studies is centered on finding the best condition to study real coevolutionary dynamics in the era of flooding new information for host-phage interactions provided by the molecular and omics technologies. Lastly, the study of phage receptors is expected to provide insight into the emergence of phage-resistance in prokaryotes (Altamirano and Barr, 2021) and further guide rational design of phage cocktail.

## Phage and Microbiome

The microbiome plays a fundamental role in our health, agriculture, fermentation, and food production (Kwak et al., 2018; Inda et al., 2019; Sutton and Hill, 2019). Existing methods to modulate the microbiome such as fecal microbiota transplantation, antibiotics, and prebiotics cause extensive shifts in microbial communities (Federici et al., 2021). These methods cannot achieve pinpoint exclusion of key species or strains of the microbial communities that are needed to maximize the targeted effects. Phages may be a viable solution to selectively manipulate microbiomes since the main components of microbiomes are bacteria, which phages due to their narrow host range can specifically eliminate target bacteria without disturbing the microbiome. Our current understanding of phage-host interactions is largely based on individual phage-host studies *in vitro*, but several studies have reported that these interactions can be different in the gut microbiome (De Sordi et al., 2017). Furthermore, the phage-mediated transfer of bacterial virulence genes (O'Brien et al., 1984; Waldor and Mekalanos, 1996) and possible influence of phage on host immune response (Sweere et al., 2019) suggest that phage therapy could be a potential risk. The ability of phages as key modulators for reshaping the microbiome is expected to be divergent depending on the surrounding environment (Sutton and Hill, 2019) that comprehensive studies to understand phage-host interactions *in vivo* are required to establish basic tools to exploit phages for safe and effective modulator of the microbial communities (De Sordi et al., 2017). Such tools that can be applied to a variety of microbial communities can unravel extensive benefits to human health, agriculture, and food industry. Although high prevalence of the uncultured microorganisms in complex microbial communities and accompanying phage diversity are challenges for phage biologist, phage application as a tool to modulate microbiome can be possible if we ameliorate our understanding on the essential role of phages in shaping microbial communities in a variety of environments *in vivo*.

## Phage Engineering

Diverse and plentiful phages can be isolated from the environment, but only a few naturally occurring phages can be useful, implying that phage engineering based on targeted genome modification of phage isolates is an attractive approach to acquire phages with improved properties. Even though various phage genome modification strategies are developed and well-established for a few phages, these engineering techniques cannot be applied for each and every phage because of the limitations of each technique (Łobocka et al., 2021). This highlights challenges in optimization and refinement of phage engineering techniques that need to address differences in the life cycle and physiology of individual phage. Another challenge in phage engineering is concerns about the release of genetically engineered phages into the environment because genetically engineered phages could have unexpected influences on bacterial community dynamics. This should be strenuously verified and considered for phage genome engineering design. Lastly other advances in phage engineering show recent progress in DNA synthesis technology allowing cell-free bacteriophage genome synthesis with reasonable cost. Unique genetics of phage can also be used to design new technologies for building novel gene circuits and scaffolds for numerous biotechnological systems (Lemire et al., 2018). Further refinement of this strategy has a great potential to revolutionize phage engineering (Yeom et al., 2020).

## Endolysin and Its Engineering

Endolysins are phage-encoded mureolytic enzymes that directly cleave bonds in the peptidoglycan of the bacterial cell wall. They are produced during the late stages of lytic phage replication cycle to disrupt their hosts, resulting in the release of viral progeny. Endolysins targeting Gram-positive bacteria have a modular structure consisting of multiple domains with diverse functions. In contrast, endolysins of Gram-negative bacteria have a globular structure with a single domain. The enzymatically active domain (EAD) determines the enzymatic cleavage of peptidoglycan and the cell wall-binding domain (CBD) is responsible for recognition of the cell surface ligands and for attraction toward its substrate. Narrow host specificity of endolysin facilitates elimination of specific target bacteria without considerable harm to the surrounding microbiome. Moreover, endolysin does not induce bacterial resistance, possibly because endolysin targets highly conserved components of peptidoglycans that are essential for bacterial viability (Fischetti, 2005). Coexistence of phage and bacteria on earth for billions of years leads us to the reasonable assumption that evolution does not allow natural endolysin to have maximum activity in order not to annihilate bacteria that are required for phage replication. Therefore, endolysin engineering is a field with many possibilities to improve endolysin and attempts such as site-directed mutagenesis and domain swapping to develop novel endolysins with upgraded features have been reported. Nonetheless, rational design for this advanced protein engineering strategy is required to improve current time-consuming and labor-intensive protein engineering processes (De Maesschalck et al., 2020). Another challenge in endolysin engineering is rational design of endolysin derivatives able to penetrate outer membrane to reach peptidoglycan layer of Gram-negative bacteria. Many efforts to design fusion endolysin consisting of hydrophobic domains of varieties of proteins or peptides with potential to penetrate Gram-negative bacterial outer membrane have established only limited success (Briers and Lavigne, 2015).

## Phage-Based Diagnostics

Rapid detection of pathogenic bacteria is crucial in minimizing the risk of bacterial diseases and food poisoning. Many of the conventional microbiological and biochemical detection methods are laborious, require skilled individuals, relatively slow, and are not always accurate. The specificity of host by phages', dependence of phage replication on bacterial metabolism, and high burst size make phages an ideal platform to develop rapid detection systems with high sensitivity, stability, versatility, selectivity, and long shelf-life (Hussain et al., 2021). Another benefit in utilizing phage for detection is the genetically engineered reporter phages detect only viable bacteria because phage can only be multiplied in viable hosts, while other detection methods detect bacteria regardless of their viability (Kim et al., 2014). A challenge with genetically engineered reporter phages however is wide application, reporter phages have a narrow host range and detection. In addition to a whole phage, phage components such as tails, tail fibers, cell wall binding domain (CBD) of endolysin can be utilized to make systems for rapid detection of bacteria. These proteins can efficiently replace antibodies because some CBDs are highly specific and their binding affinity to target bacteria is higher than antibodies used for routine diagnostics (Kong et al., 2017). Production of recombinant CBD is considerably easier compared with antibodies, further highlighting their potential benefit for diagnostics. However, despite the great benefits of utilizing phages for diagnostics, the limited availability of CBDs targeting varieties of pathogenic bacteria have been hampering their applications and further research on phage biology is needed.

## Author Contributions

The author confirms being the sole contributor of this work and has approved it for publication.

## Conflict of Interest

The author declares that the research was conducted in the absence of any commercial or financial relationships that could be construed as a potential conflict of interest.

## References

[B1] Altamirano F. Barr J. (2021). Unlocking the next generation of phage therapy: the key is in the receptors. Curr. Opin. Biotechnol. 68, 115–123. 10.1016/j.copbio.2020.10.002 33202354

[B2] Briers Y. Lavigne R. (2015). Breaking barriers: expansion of the use of endolysins as novel antibacterials against Gram-negative bacteria. Future Microbiol. 10, 377–390. 10.2217/fmb.15.8 25812461

[B3] Clokie M. R. Millard A. D. Letarov A. V. Heaphy S. (2011). Phages in nature. Bacteriophage 1, 31–45. 10.4161/bact.1.1.14942 21687533 PMC3109452

[B4] De Maesschalck V. Gutiérrez D. Paeshuyse J. Lavigne R. Briers Y. (2020). Advanced engineering of third-generation lysins and formulation strategies for clinical applications. Crit. Rev. Microbiol. 46, 548–564. 10.1080/1040841X.2020.1809346 32886565

[B5] De Sordi L. Khanna V. Debarbieux L. (2017). The gut microbiota facilitates drifts in the genetic diversity and infectivity of bacterial viruses. Cell Host Microbe 22, 801–808. 10.1016/j.chom.2017.10.010 29174401

[B6] Dion M. Oechslin F. Moineau S. (2020). Phage diversity, genomics, and phylogeny. Nat. Rev. Microbiol. 18, 125–138. 10.1038/s41579-019-0311-5 32015529

[B7] Doron S. Melamed S. Ofir G. Leavitt A. Lopatina A. Keren M.. (2018). Systematic discovery of antiphage defense systems in the microbial pangenome. Science 359:eaar4120. 10.1126/science.aar4120 29371424 PMC6387622

[B8] Federici S. Nobs S. Elinav E. (2021). Phages and their potential to modulate the microbiome and immunity. Cell. Mol. Immunol. 18, 889–904. 10.1038/s41423-020-00532-4 32901128 PMC8115240

[B9] Fischetti V. (2005). Bacteriophage lytic enzymes: novel anti-infectives. Trends Microbiol. 13, 491–496. 10.1016/j.tim.2005.08.007 16125935

[B10] Hampton H. Watson B. Fineran P. (2020). The arms race between bacteria and their phage foes. Nature 577, 327–336. 10.1038/s41586-019-1894-8 31942051

[B11] Hussain W. Ullah M. Farooq U. Aziz A. Wang S. (2021). Bacteriophage-based advanced bacterial detection: concept, mechanisms, and applications. Biosens. Bioelectron. 177:112973. 10.1016/j.bios.2021.112973 33429203

[B12] Inda M. Broset E. Lu T. K. de la Fuente-Nunez C. (2019). Emerging frontiers in microbiome engineering. Trends Immunol. 40, 952–973. 10.1016/j.it.2019.08.007 31601521

[B13] Kim S. Kim M. Ryu S. (2014). Development of an engineered bioluminescent reporter phage for the sensitive detection of viable *Salmonella* Typhimurium. Anal. Chem. 86, 5858–5864. 10.1021/ac500645c 24806327

[B14] Kong M. Shin J. Heu S. Park J. Ryu S. (2017). Lateral flow assay-based bacterial detection using engineered cell wall binding domains of a phage endolysin. Biosens. Bioelectron. 96, 173–177. 10.1016/j.bios.2017.05.010 28494369

[B15] Kwak M. Kong H. Choi K. Kwon S. Song J. Lee J.. (2018). Rhizosphere microbiome structure alters to enable wilt resistance in tomato. Nat. Biotechnol. 36, 1100–1109. 10.1038/nbt.4232 30295674

[B16] Lemire S. Yehl K. Lu T. (2018). Phage-based applications in synthetic biology. Annu. Rev. Virol. 5, 453–476. 10.1146/annurev-virology-092917-043544 30001182 PMC6953747

[B17] Łobocka M. Dabrowska K. Górski A. (2021). Engineered bacteriophage therapeutics: rationale, challenges, and future. BioDrugs 35, 255–280. 10.1007/s40259-021-00480-z 33881767 PMC8084836

[B18] O'Brien A. Newland J. Miller S. Holmes R. Smith H. Formal S. (1984). Shiga-like toxin-converting phages from *Escherichia coli* strains that cause hemorrhagic colitis or infantile diarrhea. Science 226, 694–696. 10.1126/science.6387911 6387911

[B19] O'Sullivan L. Bolton D. McAuliffe O. Coffey A. (2019). Bacteriophages in food applications: from foe to friend. Annu. Rev. Food Sci. Technol. 10, 151–172. 10.1146/annurev-food-032818-121747 30633564

[B20] Salazar K. Ma L. Green S. Zulk J. Trautner B. Ramig R.. (2021). Antiviral resistance and phage counter adaptation to antibiotic-resistant extraintestinal pathogenic *Escherichia coli*. mBio 12, e00211–e00221. 10.1128/mBio.00211-21 33906920 PMC8092219

[B21] Sutton T. Hill C. (2019). Gut bacteriophage: current understanding and challenges. Front. Endocrinol. 10:784. 10.3389/fendo.2019.00784 31849833 PMC6895007

[B22] Sweere J. Van Belleghem J. Ishak H. Bach M. Popescu M. Sunkari V.. (2019). Bacteriophage trigger antiviral immunity and prevent clearance of bacterial infection. Science 363:eaat9691. 10.1126/science.aat9691 30923196 PMC6656896

[B23] Waldor M. Mekalanos J. (1996). Lysogenic conversion by a filamentous phage encoding cholera toxin. Science 272, 1910–1914. 10.1126/science.272.5270.1910 8658163

[B24] Yeom H. Ryu T. Lee A. Noh J. Lee H. Choi Y.. (2020). Cell-free bacteriophage genome synthesis using low-cost sequence-verified array-synthesized oligonucleotides. ACS Synth. Biol. 9, 1376–1384. 10.1021/acssynbio.0c00051 32383864

[B25] Zaczek M. Łusiak-Szelachowska M. Jończyk-Matysiak E. Weber-Dabrowska B. Miedzybrodzki R. Owczarek B.. (2016). Antibody production in response to staphylococcal MS-1 phage cocktail in patients undergoing phage therapy. Front. Microbiol. 7:1681. 10.3389/fmicb.2016.01681 27822205 PMC5075762

